# Vomiting Caused by Relaxation of the Abdominal Parietes, Cured by a Bandage

**Published:** 1848-10

**Authors:** 


*Vomiting caused by Relaxation of the Abdominal Parietes cured by a bandage.*—M. Greppo reports the case of a woman whose abdominal parietes were much relaxed in consequence of repeated pregnancy, and who was troubled by habitual vomiting, which resisted all the ordinary remedies. An abdominal supporter bandage was then applied, which entirely relieved her. When, however, she neglects to wear the supporter, the vomiting returns.—*Med. Times.*

